# Transactions Texas State Medical Association: Opinions of the Press

**Published:** 1886-12

**Authors:** 


### SOCIETY NOTES.

### "TRANSACTIONS TEXAS STATE MEDICAL ASSOCIATION, 1886."

### OPINIONS OF THE PRESS.

It is a matter of no little pride to the profession of Texas to see their work so highly spoken of as in the following few extracts from our exchanges :

"The Texas State Medical Association may well be proud of its last volume of Transactions. Some improvements might be easily made in its arrangement. For example, it is difficult to understand why all the papers should be attached as an appendix, and why the publication committee did not do a little more editing. Again, should this volume go abroad some of our European colleagues will wonder why certain French and German names are not spelled correctly. The names of several of our valued contemporaries have also been well disguised in some places. There is another point to which we desire to call attention. One member of the Association reports a case of what seems to have been chronic catarrhal gastritis with some unusual symptoms. It is reported as "A Remarkable Case in my Practice." The first thing to do in writing an article is to give it a proper and descriptive title. We call attention to this now because it is a fault of so many writers to make a title which is descriptive of anything from a ruptured perineum to hay fever. "Cases from Practice" is an abomination which is too frequently seen. The title of a paper should be indexical of the whole paper. All writers may not know this, but a publication committee is supposed to know it.

For all this, however, the volume under consideration is one upon which the members of the Texas Association may congratulate themselves. It is very handsome, and well printed on excellent paper. It contains some good articles, which would be all the better if they were properly indexed. Six hundred and ninety-one pages of book is a good deal to send out without an index. One of the best papers in the volume is on "Boracic Acid in Surgery and Gynecology," by Dr. C. M. Ramsdell, of Lampasas, which will repay careful reading. Dr. O. L. Williams, of Chapel Hill, reports two cases in which electricity acted well in congestive fever (pernicious malarial?). Dr. Q. C. Smith, of Austin, in an interesting paper on the "Treatment of Sporadic Dysenteries," very properly condemns opium, mercury and starvation. Dr. T. C. Osborn, of Cleburne, calls attention to the usefulness of bimuriate of quinine with urea generally, and particularly in a case of pneumonia. Dr. J. R. Briggs, of Fort Worth, calls attention to the value of lycopus Virginicus (bugle weed) in the bites and stings of insects and reptiles. This plant seems worthy of a closer examination than has been given to its physiological action. A valuable contribution to the subject of malaria may be found in a paper by Dr. Henry K. Leake, on "Malarial Irritation of the Bladder in the Female." Those who may be inclined to doubt the wisdom of conservative treatment in gun-shot fracture of the femur would do well to read the report of four cases, all of which recovered, by Dr. J. T. Field, of Fort Worth. Dr. J. G. O'Brien, of Dublin, reports a case of extraction, by tracheo-laryngotomy, of a watermelon seed from the air passages of a child 12 months old, with recovery. Dr. B. F. Brittain, of Jacksonville, records a very curious case of necrosis of the lower maxilla, which began with toothache. The necrosed portion was removed, with a good result. Dr. C. M. Farrar, of Fort Worth, reports a case of amputation of both legs, with recovery, and death subsequently from abscess of the pancreas. Dr. O. Eastland, of Wichita Falls, reports a case of dislocation of the long head of the biceps, which was reduced spontaneously on the fourth day, after ineffectual efforts by the method of Prince. The Prize of the Association was awarded to Dr. J. R. Briggs, of Fort Worth, for a paper on "Physical and Mental Culture," which we hope to notice soon in another department."—*Journal American Medical Association.*

---

THE SOUTHERN PRACTITIONER, of Nashville, says:

The Texas State Medical Association and its committee of publication are to be congratulated on one of the handsomest and most elegant volumes of Society Transactions that we have ever seen. As an outcome of the malarial belt, it is rather a "stunner," and will compare more than favorably with those from more intellectual(?) localities on this continent, or even "Yurrup."

The handsome cloth binding, elegant paper and neat typography would be a credit to the most enterprising and completely equipped publishing house of the most metropolitan city of either continent. But Texas is a great State, with great doctors, and a future that will yet prove marvelous.

We regret exceedingly that we have not space to give a full and extended review of tne subject-matter contained in the elegant volume before us. A brief examination convinces us, that from the minutes of the meeting, and the President's address, to the very full and comprehensive index, it is teeming with good things, and will furnish mental pabulum for many a good feast, prepared by the very progressive and talented members of the medical profession in Texas.

---

THE WEEKLY MEDICAL AND SURGICAL REPORTER, of Philadelphia, says:

This portly volume of nearly seven hundred pages, well printed and carefully edited, proves in the completest manner that the physicians of Texas are behind no others in their devotion to the progress of their science. The reports of the chairmen of the various sections on the progress of their respective branches are prepared with care, and in each section there are quite a number of original communications from physicians in the State. Nearly all of these contain valuable matter, and deserve preservation in the volume. It is, in all respects, a meritorious publication.

---

And the NEW YORK MEDICAL JOURNAL says:

The "Transactions of the Texas State Medical Association" at its eighteenth annual session, held at Dallas on the 27th, 28th, 29th and 30th of April, 1886, have just been issued in the form of a handsome volume of nearly seven hundred pages. The paoers read at the meeting are for the most part very meritorious, and, in particular, the reports made to the various sections strike us as of more than ordinary value. We regret to learn that the Secretary died during the preparation of the volume, but we congratulate the Association on the appointment of so energetic and capable a successor as has been found in Dr. Daniel, of Austin.

The North Texas Medical Association held its annual meeting at Paris, Texas, on the 14th, 15th and 16th insts., Dr. F. D. Thompson, of Sherman, the president, presiding. In our next we hope to be able to give a report of the work done. Judging from the names of the chairmen of sections, we expect something good.

